# Perceptual Phenomena Cannot Be Approached from a Single Perspective

**DOI:** 10.3390/jintelligence11110214

**Published:** 2023-11-10

**Authors:** Alessandro Soranzo, Luca Taddio

**Affiliations:** 1 Centre for Behavioural Science and Applied Psychology (CeBSAP), Sheffield Hallam University, Sheffield S1 1WB, UK; 2 Dipartimento di Studi UManistici e del Patrimonio Culturale, University of Udine, 33100 Udine, Italy; luca.taddio@uniud.it

**Keywords:** phenomenology, neurophysiology, stimulus error, experience error, mind/body dualism, ambiguous expressions, theory of levels of reality

## Abstract

This article explores the relationship between neurophysiology and phenomenology in the context of ambiguous figures. Divided into three parts, the study investigates new forms of stimulus and experience errors that arise from ambiguous figures. Part 1 discusses the limitations of a single-disciplinary approach and cautions against relying only on neurophysiological explanations for perceptions. A sole reliance on neurophysiological explanations can lead to stimulus and experience errors, as well as to the development of an unfounded mind/body dualism. Part 2 focusses on the stimulus error associated with ambiguous figures. It also shows how the Mona Lisa’s ambiguous expression can cause the experience error. Unlike other forms of ambiguous figures, different expressions of Mona Lisa are perceived when seen in different definitions. It is shown how assigning a higher ontological status to one of the expressions because it aligns with our knowledge of the nervous system, as conjectured by some authors, gives rise to the experience error. Part 3 emphasises the importance of complementing neurophysiological interpretations with phenomenological ones for a better understanding of perceptual phenomena. Phenomenology provides constraints and corrections to neurophysiology, whereas neurophysiology informs phenomenology through empirical findings. The theory of levels of reality is introduced as a framework to underlie the connections and dependencies between different perspectives. Using both neurophysiological and phenomenological approaches, a comprehensive understanding of perceptual phenomena emerges, surpassing the limitations of each discipline. This method encourages a holistic view of perception, where neurophysiology and phenomenology coexist, complementing and enriching each other’s insights.

## 1. Introduction

This article emphasises the importance of a multi-disciplinary approach in the interpretation of perceptual phenomena, particularly when examining ambiguous figures.

Science, driven by its quest to comprehend natural phenomena, faces the challenge of deciphering complex and ever-changing perceptions that are dynamic, contextual, and relational (Lederman et al. 2013). Consequently, exploring these phenomena from diverse and sometimes conflicting perspectives becomes paramount, as advocated by Rittel and Webber (1973).

In contrast, the philosophy of science posits that advancements in perception should exclusively stem from physiological research (Churchland 1989; Churchland and Sejnowski 1992). Authors have even advocated for the replacement of psychological language with physical language, encompassing behaviour and brain states (Hempel et al. 1949).

According to this reductionistic approach, perceptual phenomena must be reduced to their elementary stimulations in isolation, preventing us from recognising their configurational characteristics.

This article challenges such a singular approach. In particular, it aims to demonstrate that explaining the nature and meanings of phenomena solely in reductionistic terms of neuronal correlates is fundamentally flawed; ultimately, this is a categorically incorrect endeavour.

The subjective character of experience eludes capture through any reductive analysis of the mental (Nagel 1974). A comparative analysis among diverse methodologies illuminates the connections and potential laws of interdependence across different perspectives, often serving as the drive for the development of these enquiries themselves (Albertazzi 2013).

The initial section of this article unveils the limitations of a single-disciplinary approach and cautions against the pitfalls of relying solely on reductionistic explanations to account for perceptions. Part 2 subsequently presents a case study on ambiguous figures, underscoring the aforementioned limitations. Finally, Part 3 outlines the advantages that emerge from adding to the neurophysiological approach the phenomenological one.

## 2. Part 1: Limits and Perils of Investigating Perceptual Phenomena from a Single Perspective

The argument advocating that advancements in perception should solely rely on neurophysiological research, as claimed by Churchland and Sejnowski (1992), implies that visual phenomena are constructed from raw sensations. Gilchrist (2022) argues that this perspective leads to an unsupported distinction between raw sensation and perception, resulting in a mind/body dualism, attributing a portion of vision to the body and the rest to the mind. Such dualism conceals subtle sources of stimulus and experience errors, which we will briefly describe next and further explore in Part 2 in relation to ambiguous figures. Moreover, the notion itself of perception occurring in distinct stages poses inherent problems, as we shall discuss in the concluding section of Part 1.

### 2.1. Stimulus Error

**Figure 1:**
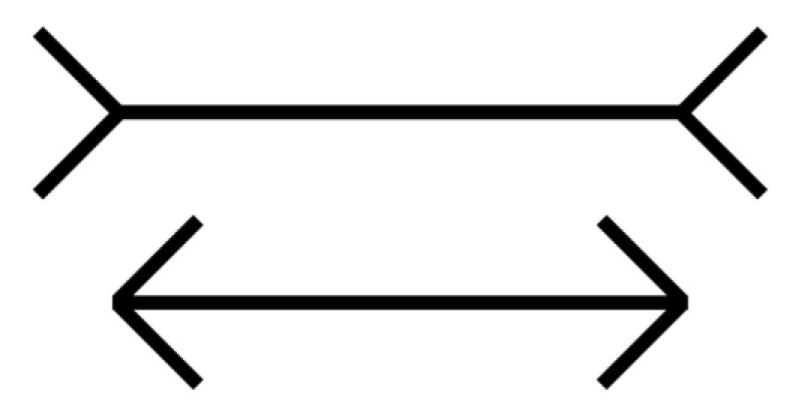
Müller-Lyer illusion.

Köhler (1929) defines the stimulus error as the “danger of confusing our knowledge about the physical conditions of sensory experience with this experience as such” (p. 162). For example, saying that the two lines in the Müller-Lyer illusion (Müller-Lyer 1896), shown in Figure 1, are of the same length is a prototypical example of the stimulus error.

When we assert that the Müller-Lyer illusion shows lines of the same length “in reality”, it is the language that is misleading: it seems that we are indeed measuring what we are observing, the Müller-Lyer illusion, while instead, we are just measuring the two lines within the flankers, not the full configuration. The measurement of the two lines implies a reduction in what has actually been experienced. The measurement does not include the flankers, which are the real determinants of the illusion. Using a ruler on the two lines does not measure the Müller-Lyer illusion, but rather, just the length of the two lines. However, the two lines by themselves not only share the same length, but also appear to be the same length.

The measurement of the lengths of the lines in the Müller-Lyer illusion is a simplification of what happens in our phenomenological world. Measuring the illusion, instead, would mean measuring whether the lines appear more or less dissimilar by, for example, bending the flankers. But a new measure, with a version of the Müller-Lyer illusion with bended flankers, cannot be obtained using a ruler, but rather, through our phenomenological experience. Saying that the two lines in the Müller-Lyer illusion are of the same length is therefore a prototypical example of the stimulus error because we confuse what we know with what we experience (Taddio 2013, 2022).

Although we referred to the stimulus error in singular form, Bozzi (1972) specified that it manifests itself in various ways, depending on the physical conditions of sensory experience at which perception becomes confused. A percept can be confused with characteristics of the distal stimuli, of the proximal stimuli, or of the constellation of stimuli. Confusing one or a combination of these stimuli with the percept gives rise to different variants of the stimulus error (see also Taddio 2011). Legrenzi (1998) demonstrates the far-reaching impact of this error beyond the field of the psychology of perception, extending into other scientific domains, such as the psychology of reasoning.

### 2.2. Experience Error

The experience error complements the stimulus error and involves attributing to the senses properties that pertain instead to the phenomenal experience. In his work, Köhler (1929) proposed the following: “As I see it, another mistake [to the stimulus error], which I propose to call the experience error, is just as unfortunate. This error occurs when certain characteristics of sensory experience are inadvertently attributed to the mosaic of stimuli” (p. 95). For example, asserting that colours reside in objects is a form of experience error because colours are a creation of our visual system (Pomerantz 2014).

The second part of this article will examine how exaggerated faith in the physiological perspective can result in both stimulus and experience errors.

### 2.3. Problems with Dualism

Dualism poses several problems, due to its inadequate explanation of the intricate nature of perceptual phenomena and its failure to acknowledge the interconnectedness between the mind and body. Ben-Zeev and Strauss (1984) emphasise that the assumption underlying dualism, which suggests that visual perception involves an early sensory stage driven local retinal stimulation (performed by the body), followed by the interpretation of those sensations (performed by the mind), implies a distinction between sense reception and sense perception. According to this distinction, sensations are meaningless, while perceptions have meaning. The transition from meaningless sensations to meaningful perception is intended as follows: sensations are the result of physical causes without active mental contributions to their creation. In contrast, meaningful everyday perceptions heavily involve mental (primarily cognitive) processes (Rittel and Webber 1973). However, this argument encounters significant problems. First, the postulation of a pure mental state lacks empirical support. A pure mental state cannot be a state within a system that is not influenced by the nature of that system. This means that there cannot be a mental state unaffected by the typical features of one’s mind.

In the dualistic approach, sensation is characterised as a mental response, yet the nature of the response system, which comprises one’s knowledge, memory, will, emotion, expectations, and more, is assumed not to play a role in determining that response.

This assumption stands out as peculiar. According to the dualistic approach, pure sensation should be devoid of any relationship or meaning, akin to an isolated sensory atom or a chaotic flux. However, even in these cases, it is difficult to comprehend how sensations can exist without some form of relationship.

Another problem with dualism lies in its inability to explain how a meaningful perception can emerge from a meaningless sensation. The fundamental challenge is understanding how the mind can create a meaningful world from completely meaningless materials.

The objection to pure sensation extends beyond theory to empirical observations. There is no empirical evidence supporting a non-meaningful sensory stage. Every sensory quality proposed to be part of that stage has been found to be “contaminated” with meaning. It is impossible to locate a reception room in a mental system where qualities are passively registered and await processing (Ben-Zeev and Strauss 1984). Even the eminent neurophysiologist Charles Scott Sherrington admits that the “mind rarely, probably never, perceives any object with absolute indifference, that is, without ‘feeling’. In other words, affective tone is the constant accompaniment of sensation” (Sherrington 1900, p. 974). In agreement with Köhler (1929), we conclude that perception is a unified process that responds to an extended pattern of stimulation.

This first part of this article demonstrated the limitations of exclusively approaching perceptual phenomena from a physiological perspective. To illustrate the benefits of embracing a multidisciplinary approach that incorporates phenomenology, Part 2 delves into the ambiguous stimuli.

## 3. Part 2: Case Study of Mona Lisa’s Ambiguous Expression

**Figure 2:**
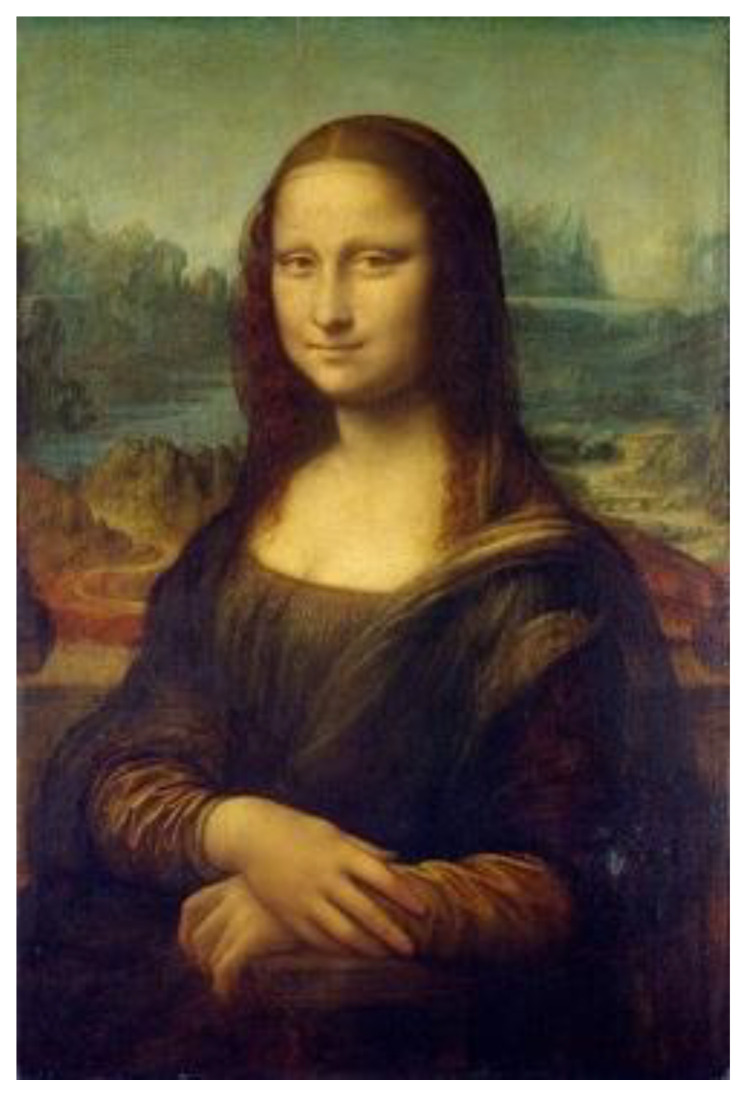
Mona Lisa 1503-6, Louvre.

One of the most known ambiguous stimuli is the Mona Lisa’s ambiguous expression (Figure 2).

Ambiguity arises from the fact that, like an animated subject, Mona Lisa appears to change expression before our eyes. This is how the art historian Gombrich (1995) described this perceptual phenomenon: “Sometimes [the Mona Lisa] seems to mock at us, and then again we seem to catch something like sadness in her smile” (p. 219).

Gombrich (1995) suggested that this expression change occurs through *sfumato*. From the Italian word for vanishing like smoke, in *sfumato*, the transitions from bright to dark, or from one colour to another, are subtle, to soften or obscure sharp edges. This technique was originally developed by northern European oil painters such as Jan van Eyck (1390–1441), in which a translucent paint is laid over an opaque one. Hence, it generates an overlaying of multiple translucent layers of paint (Elias and Cotte 2008). In his *Trattato della Pittura*, Leonardo describes *sfumato* as without lines or borders, in the manner of smoke or beyond the focus plane, thus trying to create visually indistinguishable passages from one colour to another.

### 3.1. Neurophysiological Interpretation of Mona Lisa’s Ambiguous Expression

Livingstone (2000) interprets the Mona Lisa’s ambiguous expression considering that *sfumato* generates an overlap of different spatial frequencies. Spatial frequencies refer to the level of visible detail at a given visual angle, with high spatial frequencies representing minute details and low spatial frequencies representing coarse aspects of the image (De Valois and De Valois 1980). Livingstone’s explanation is fundamentally neurophysiological because it focusses on the selective sensitivity of retinal receptors to different spatial frequencies. Cones, concentrated in the central area of the eyes, are sensitive to high spatial frequencies (minute details), while rods, situated in the peripheral area, are sensitive to low spatial frequencies (coarse resolution). The simultaneous presence of overlapping spatial frequencies through *sfumato* creates a smile that emerges predominantly when viewed from the periphery of the eye, where only low spatial frequencies are available. On the contrary, when the mouth is seen from the gaze centre, where high spatial frequencies are also visible, the smile fades.

From what is said in Part 1, it is evident that this explanation falls into the category of the stimulus error, a prototypical example of confounding knowledge about the physical conditions of sensory experience (the distribution of retinal receptors) with the experience itself (the perception of an ambiguous expression). There is another issue that arises indirectly from this interpretation. It clarifies the systematic nature of the phenomenon: it is the visible details that determine the perceived expression, not the viewer’s state of mind or imagination. Once the systematic nature of the phenomenon has been clarified, the lifelike quality of the Mona Lisa itself “might not be so mysterious after all” (Livingstone page 82). However, we believe that the mysterious nature of the portrait is one of the key elements contributing to its artistic value. As outlined by Gombrich (1995), the Mona Lisa expression appears “rather mysterious, and so it is; that is the effect of every great work of art” (p. 219). According to Gombrich, great art should evoke mysterious feelings in the viewer and engage them on a deep level. Mystery invites contemplation and interpretation, thus deepening the viewer’s engagement with the artwork. As Livingstone maintains, the neurophysiological explanation reduces the mystery of the work, thus reducing its artistic appeal. This reduction diminishes the sense of wonder and limits the room for interpretation, diminishing the overall experience of engaging with the artwork.

Thus, Livingstone (2002) deserves credit for acknowledging the ontological limitations of the neurophysiological explanation of aesthetics. By reducing the Mona Lisa’s expression to a causal process of sensory transmission from retinal neurons, the neurophysiological approach disregards the significance of the overall configuration (Verstegen 2005). However, the configuration adds complexity and emotional depth to the phenomenon, highlighting the necessity of complementing the neurophysiological interpretation with a phenomenological one (Hatfield 1990, 2000).

### 3.2. Phenomenological Interpretation of Mona Lisa’s Ambiguous Expression

As in neurophysiology, *sfumato also* plays an important role in Mona Lisa’s ambiguous expression in phenomenology, but it is conceptualized differently. The phenomenological perspective emphasises how *sfumato* alters the appearance by modifying the perception of their borders.

Building upon Katz (1911)’s influential work, Kanizsa (1954, 1979) examined how the modality of the appearance of colour changes depending on the type of border it possesses. The author observed that gradual colour transitions, such as those achieved through *sfumato*, give rise to what Katz termed “film colours.” These colours exhibit a quality that allows sight to seemingly penetrate them, akin to the sky or fog. On the contrary, the “surface colours” appear opaque, compact, and solid. In the case of the Mona Lisa, the outlines of the mouth exhibit a softening effect, with no clear demarcation between the mouth and the surrounding facial areas.

The slightly darker smudges over the corners of Mona Lisa’s mouth appear either shadowy or mouth-like depending on the visible details, gaining the designation of “ambiguity smudges” (Soranzo, forthcoming). When minute details are discernible, a boundary between the mouth and the ambiguity smudges become visible, resembling a cast shadow on the cheek. However, when minute details are less distinct, the boundary becomes imperceptible, causing the smudges to be perceived as part of the mouth because of the gestalt principle of good continuation. In this case, the shadow seems to continue the upward curvature of the lips, contributing to the impression of a subtle smile 1.

In summary, *sfumato* plays a pivotal role in both neurophysiology and phenomenology, although with differing conceptualisations. In neurophysiology, it generates ambiguity right from its inception at the retinal receptors. In phenomenology, on the other hand, *sfumato* exerts an indirect effect: it first alters the mode of colour appearance, and then, its effects propagate throughout the perceptual system, culminating in ambiguity by modifying perceptual belongingness.

The divergence between neurophysiology and phenomenology can be further explored by examining how these approaches attribute ontological significance to the emotional state conveyed by Mona Lisa.

### 3.3. The “True” Mona Lisa Expression

Livingstone (2002) points out that “our ability to correctly interpret facial expressions in general is better in our peripheral vision than in the center of gaze. […] images or movies of people that mimic the blurring effect of peripheral vision might aid in judging their *true* emotional state…” (p. 83). These words seem to indicate that Livingstone advances an ontological classification of the emotional states visible in the Mona Lisa, attributing priority to the one seen with the periphery of the eye, the cheerful one. Apart from the possible interpretations of this sentence, it signals a general tendency towards ontological realism that emphasises objects over perceptions.

Certainly, empirical evidence that expressions can be successfully identified at coarse resolution exists (however, it does not appear that this identification can be superior to high-resolution identification). For instance, Smith and Rossit (2018) and Bayle et al. (2011) found that certain emotions, particularly happiness, can be recognised through peripheral vision, while Smith and Schyns (2009) discovered that we possess the ability to properly identify emotions even from a distance (distance reduces spatial frequencies, similar to peripheral vision).

From the perspective of subjective experience, attributing a higher ontological status to the expression perceived at low spatial frequency (coarse resolution), as Livingstone (2002) seems to imply, lacks meaningfulness. Phenomenologically, both expressions possess the same status. This can be exemplified considering the well-known example of the broken stick in the water; the stick visually appears broken and will always appear to us that way whenever we observe it in the water; such are the properties of those two things (stick and water) in that particular relationship.

At most, we may question which of the expressions—contentment or melancholy—holds greater ontological value for our subjective experience. It will be shown that this is not the one seen in coarse resolution.

Before we dive into this matter further, two clarifications are necessary. The first pertains to the types of ambiguous expression that can be perceived in portraits, while the second addresses ambiguous figures and how they can give rise to stimulus and experience errors.

### 3.4. Types of Ambiguous Expressions

As a difference from other types of ambiguous figures, Mona Lisa’s expression is a multi-stable stimulus. Like Schrödinger’s cat being simultaneously dead and alive, the expression of the Mona Lisa can be seen as both content and melancholic, existing in a superposition of states, until it is viewed by an observer. When this happens, the superposition collapses into one or another of the possible expressions.

**Figure 3:**
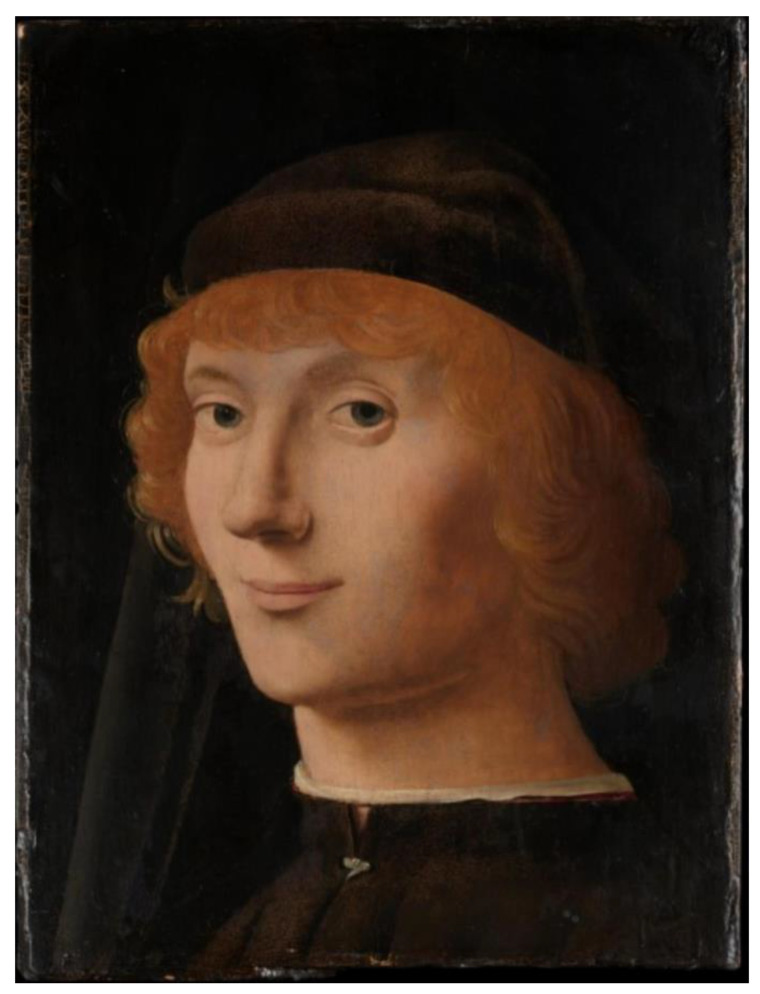
Portrait of a Young Man by Antonello da Messina (c.a. 1470). Metropolitan Museum of Art.

It is important to note that this superposition is distinct from being merely a midpoint between contentment and melancholy. To better appreciate this distinction, we can examine the portrait of a Young Man by Antonello da Messina (Figure 3).

House (2018) suggests that Antonello da Messina introduced the subtle smile in his artwork, such as that one shown in Figure 3, to convey the inner life of the sitters. The author argues that this approach predates Leonardo da Vinci’s dictum of representing ‘moti mentali’ (mental states) in portraits. However, it is important to note that Antonello’s smiles differ in ambiguity compared to Mona Lisa’s. In Figure 3, the expression remains consistent regardless of viewing conditions, consistently conveying a midpoint between contentment and melancholy. In Schrödinger’s analogy, the cat is moribund.

Mona Lisa’s expression belongs to the ambiguous figures that are multi-stable, such as Rubin (1915)’s vase/profiles, which we are going to explore next.

### 3.5. Ambiguous Figures and the Stimulus Error

Ambiguous figures present visual information that can be perceived in multiple ways, leading to different interpretations or perceptions. These ambiguous figures can lead to unique types of stimulus error and experience error. By exploring and understanding these errors, we can gain further insight into the complexities of perception and the subjective characteristic of our experiences.

Although Bozzi (1972, 1998) and Taddio (2011) identified a number of varieties of stimulus error based on the physical or physiological mechanisms with which the percept is confused, their analysis did not specifically consider ambiguous figures. When analysing ambiguous figures, additional varieties of stimulus and experience error can be distinguished, arising from the nature of these stimuli.

**Figure 4:**
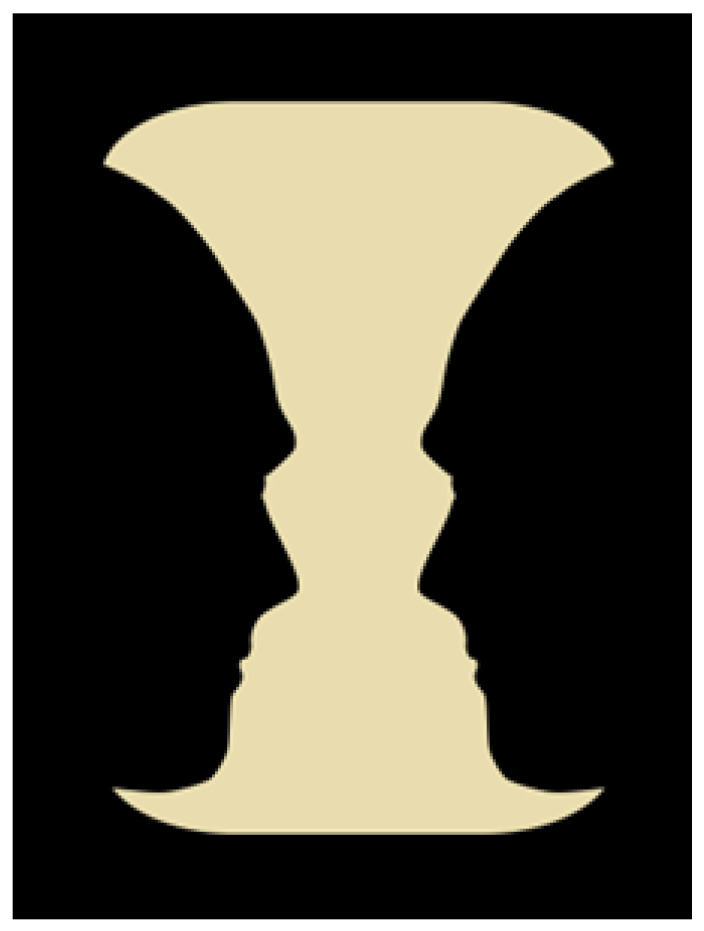
Rubin vase/profiles.

The first variant involves assigning a higher ontological value to one of the knowledge-based percepts. It can be illustrated using Rubin (1915)’s vase/profiles configuration (Figure 4).

Imagine discovering Rubin’s lost diary. According to the diary, Rubin worked on the figure, wanting to draw a vase. It was late at night, and he left it unfinished due to fatigue, with the goal of finishing it the next morning. When he returned to his artwork the following day, he realised that the figure could also be perceived as two profiles facing each other.

Asserting that there is a vase in the figure because Rubin intended to depict it is a variant of the stimulus error arising from knowledge of the figure’s history. Another variant of the stimulus error involves assigning an equal ontological value to two percepts when one should have precedence over the other.

**Figure 5:**
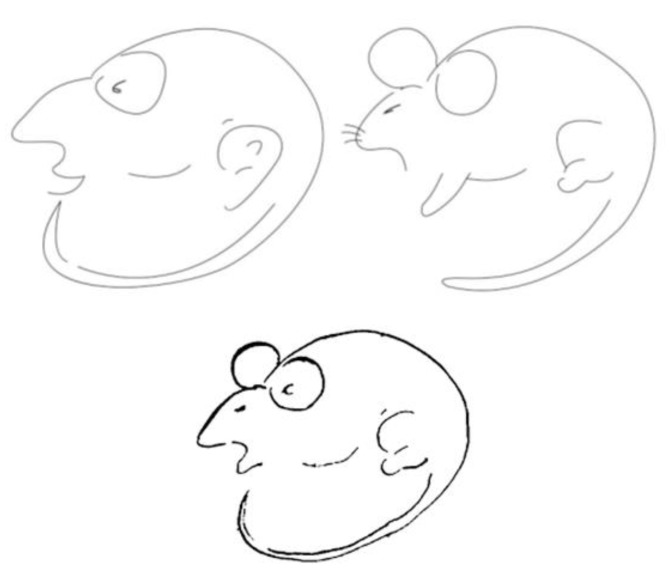
(**Top**) Adaptation figures. Unambiguous man (**left**), unambiguous rat (**right**). (**Bottom**) Rat/man figure (Bugelski and Alampay 1961).

To illustrate this variant, consider the bottom of Figure 5, where two percepts are detectable: a rat and a man. Research by Bugelski and Alampay (1961) demonstrated that prior exposure to an unambiguous image of a man or a rat (top-left and top-right of Figure 5, respectively) influenced the perception of the ambiguous figure. When participants were exposed to the unambiguous man, they were more likely to perceive the ambiguous figure as a man, while exposure to the unambiguous rat led to the perception of a rat. This shows how prior exposure to a related stimulus can influence subsequent perception.

In this context, asserting to see both a man and a rat after being exclusively exposed to one of them is a variant of the stimulus error. This error arises from the knowledge that prior exposure affects the identification of the figure.

### 3.6. Ambiguous Figures and the Experience Error

As mentioned, Livingstone (2002) attributed to the merry expression of Mona Lisa a higher status. We claim here that this attribution contrasts with our subjective experience. This attribution does not fit either of the stimulus error variants above exposed. The Mona Lisa’s expression differs from the two ambiguous figures described above in that different percepts originate from different proximal stimuli. The “content” Mona Lisa appears in coarse resolution, while the “melancholic” Mona Lisa appears in high resolution. These are distinct proximal stimuli.

Therefore, stating that the true Mona Lisa expression is the one viewed in low resolution represents a form of the experience error, not of the stimulus error.

Examine the following scenario: In a window shop, you notice a pullover that appears red from a distance. Upon approaching the shop, it appears to be an orange pullover. What is the true colour? Although you have experienced two percepts, you would likely trust what you saw up close and in higher resolution. And you probably would not buy that pullover if you wanted it red because, for you, the true colour is orange.

The problem here is due to the relationship between our body, understood as a perceptual system, and reality; this relationship establishes a link between the “world” and our “subjectivity.” The distinction between what is “internal” and what is “external” implies a consciousness that can perceive and localize phenomena. Our immediate experience is, from an ontological standpoint, emphasised by perception. To dive deeper into this matter, it is essential to understand the entire process. Thus, what we refer to as the “world” and the “subject” are merely experiential poles emerging from the process itself, arising from the exchange of information between the body, seen as an integrated physical system, and the surrounding physical environment. It becomes a matter of exploring phenomena within their corresponding levels of complexity; in this sense, the perceived world cannot be reduced to mere physical stimulation, but is a complex emergent phenomenon. This complexity of the perceived world and of its objects also applies to the Mona Lisa’s expression. From a distance, Mona Lisa appears content, but when observed up close, she looks melancholic (Soranzo and Newberry 2015). As we tend to place more trust in what we perceive in higher resolution, the melancholic state holds a higher ontological status in our subjective experience. Assigning greater ontological status to what is perceived at low resolution, as Livingstone (2002) did, because it aligns with our knowledge of the nervous system, falling within the definition of the experience error (Köhler 1929). Indeed, Livingstone attributed to the proximal stimuli properties that are attributable only to perception.

The argument that we place more trust in what we perceive in higher resolution is supported by the empirical findings of Gloriani and Schütz (2019). The authors investigated the scotopic foveal scotoma, which is the rod-free zone around the fovea in the visual field. Under scotopic conditions, this region lacks photoreceptor stimulation, leading to a scotoma (Curcio and Allen 1990). Gloriani and Schütz (2019) found that under scotopic conditions, the scotoma in the fovea is filled with information from the immediate surroundings. Most interestingly for our argument, the authors found that we tend to trust this inferred information more than veridical information from the periphery of the visual field. Remarkably, a similar preference for foveal information emerged even under daylight illumination, indicating a default preference for foveal information, at high resolution, even when it is not accurate.

It should be noted that while these findings support trust in foveal information, Hubel (1997) reported apparent discontinuity in a straight line passing through the fovea under monocular scotopic conditions, with a 1 ° gap. However, this observation was based on the author’s personal experience, and it is not supported by empirical studies. Indeed, we could not see this supposed discontinuity of straight lines seen in monocular scotopic vision. Both Bozzi (1972) and Taddio (2011) reported a very similar circumstance as a prototypical example of the stimulus error. Excessive reliance on neurophysiological facts may have biased Hubel’s interpretation of the line.

In summary, while a neurophysiological approach assigns to the Mona Lisa’s expression of contentment a higher ontological status, from a phenomenological point of view-that considers the subjective nature of experience, it is the “melancholic” expression that takes precedence because we trust more what is seen at high resolution.

## 4. Part 3: Synergy between Neurophysiology and Phenomenology

The concluding part of this article explains the advantages of using both the neurophysiological and the phenomenological approaches to interpret perceptual phenomena.

As highlighted by Todorović (1987), phenomenology often informs neurophysiology by providing constraints within which neural activity can be explored. Furthermore, phenomenology can even correct neurophysiology. For example, the neurophysiological explanation of lightness contrast (the phenomenon whereby a grey surface looks darker on a light than on a dark background), based on retinal receptor interactions, was corrected by Agostini and Galmonte (2002).

**Figure 6:**
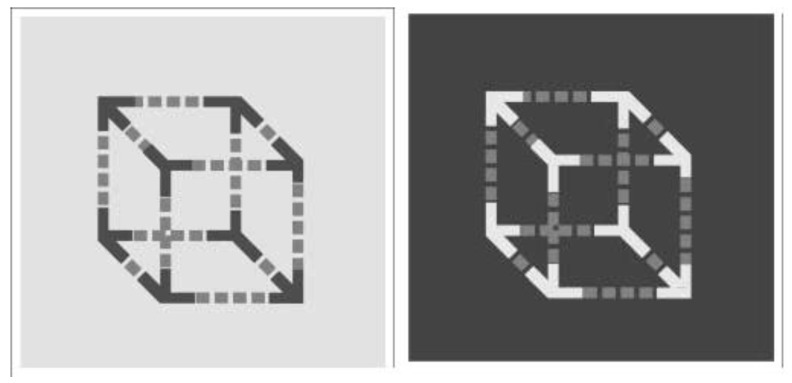
The grey dashed lines to the left appear lighter than those to the right even though their background is lighter (Agostini and Galmonte 2002).

In the case of the configuration depicted in Figure 6, the grey dashed lines on a light background (shown on the left), perceptually belonging to dark corners, appear lighter than equal greys on a dark background, belonging to light corners, shown on the right of Figure 6. The neurophysiological explanation based on retinal lateral inhibition, where receptors stimulated by lighter surfaces inhibit neighbouring receptors, is challenged by this evidence. Neurophysiologists need to explore alternative explanations to account for the lightness contrast phenomenon.

However, in some circumstances, it is neurophysiology that informs phenomenology. For example, physiological studies suggest a functional distinction between the ventral and dorsal pathways of the visual stream. Neurones within the ventral stream, which runs from the occipital cortex to the temporal cortex, respond selectively to visual features relevant for object identification, such as colour, shape, and texture (the “what” stream). Dorsal stream neurons, which travel to the parietal cortex, respond selectively to spatial aspects of stimuli, such as the direction and speed of stimulus motion (the “where” stream; Ungerleider and Pessoa 2008). These physiological insights inspired researchers to assess whether vision for perception and vision for action are dissociated (Goodale and Milner 1992; Franz et al. 2000; Haffenden et al. 2001). Studies on the Müller-Lyer illusion (Figure 1) have shown that actions such as transporting the hand from one end to the other end of the segment may not be biased by the illusion, showing a dissociation between vision-for-perception and vision-for-action (Bruno et al. 2008).

This example is revealing because neurophysiological studies indicate the characteristics that the phenomenon should possess. By moving the hand from one end to the other end of the segments, we measure the Müller-Lyer illusion, similar to what we do when using a ruler. However, measuring the Müller-Lyer illusion with a ruler or with the hand is an inappropriate endeavour, as the configuration includes the appendices; the illusion can be measured only with the eyes, not with a ruler or the hand (Taddio 2022).

This suggests that the same phenomenon needs to be investigated on different levels of reality (Poli 2006), each with its own autonomy and independence. The next section delves into the meaning of reality levels.

### Levels of Reality

The theory of levels of reality provides a natural framework conducive to the development of a nuanced theory of causal dependence. This framework posits that reality exhibits a multilayered structure, manifesting depth within both our individual consciousness and in the external world. The lower levels of reality serve as a foundational necessity for the existence of higher levels, yet the comprehensive explanation of the latter cannot be entirely reduced to the former. For instance, while the presence of cells within our bodies is indispensable for the proper functioning of organs, the field of physiology cannot be wholly subjugated to the realms of cytology and histology (Pilgrim 2019). Similarly, though phenomenology undeniably relies upon physiological underpinnings, its own configuration and governing principles maintain a level of autonomy distinct from the framework and laws governing physiology.

The attribution of distinct ontological categories to each family of processes, whether phenomenological or physiological, does not diminish their ontological legitimacy. Furthermore, the presence of dependency relationships characterizing the interplay between phenomenology and physiology does not render the dependent elements any less real than what the former depends on (Zhok 2022).

By adopting the theory of levels of reality, we gain valuable insights into the intricate interconnections and dependencies existing between these distinct perspectives (Feest 2021; Kubovy 2001) while preserving their individual areas of emphasis. This amalgamation of approaches allows for a comprehensive and nuanced comprehension of perceptual phenomena, transcending the confines of any single-disciplinary perspective.

Nevertheless, certain limitations inherent in the theory of levels of reality need to be acknowledged. Varzi (2013) cautions against the potential fallacy of imposing a strictly stratified view of reality, as it might entail projecting our own cognitive frameworks onto the external world. Therefore, it is prudent to conceive of the notion of “level” within the context of an emergentist perspective, not as an outright “theory”, but rather, as a methodological and epistemological approach. In this sense, the concept of “level” functions as a tool for the analysis of the relational emergentist.

An emergentist perspective is invaluable for comprehending and scrutinising the diverse levels of reality. It implies a commitment to “naturalistic monism” and primarily serves as a counterpoint to dualistic perspectives (see Part 1 of this article). The ontological framework that emerges from this perspective accords a privileged epistemological status to the domain of science, granting it the authority to formulate predictions while remaining open to the possibility that descriptive categories extending beyond the physical realm are equally fundamental (Zhok 2022).

Emergent properties, as a cornerstone of this perspective, manifest themselves as attributes of wholes that arise from the intrinsic characteristics of their constituent parts. The term “property” here extends beyond mere quality or attribute, encompassing a set of consequences and implications inherently tied to an entity. An emergent property distinguishes itself from its underlying properties, which are subvenient, while maintaining a complex relationship with their existence. In other words, an object should be described as an organisation of parts connected by relations, and having properties that parts do not have; such properties are called “emergent” or “second level” (Urbani Ulivi 2019).

Moreover, emergent properties exhibit irreducibility to the properties from which they arise. Thus, knowledge of subvenient properties does not provide a deterministic blueprint for emerging ones. To illustrate this, the perception of the Mona Lisa, whether viewed peripherally or at its centre, does not conclusively determine the resultant perceived expression, as empirically demonstrated by Soranzo (Soranzo, forthcoming).

In conclusion, the examination of ambiguous figures highlights the intricate relationship between neurophysiology and phenomenology. While neurophysiology offers insights into the underlying mechanisms and neural processes, it is unable to serve as rock-bottom description of perceptual reality. Phenomenology provides subjective experience, a meaningful interpretation of ambiguous figures, and, in the context of art, a reflection of aesthetic appeal.

By integrating these two perspectives, we can enrich our understanding of perceptual phenomena. The interplay between neurophysiology and phenomenology allows us to uncover connections and dependencies at different levels of reality. Although they have separate foci, their interconnectedness unveils a more comprehensive and nuanced understanding of perception. As we embrace the complexities of perceptual phenomena, it is essential to acknowledge the distinct contributions of both approaches. We must utilize their synergy to further our understanding. This synergy not only enhances our scientific endeavours, but also encourages a holistic and enriched appreciation of the human experience.

## Data Availability

Not applicable.

## Note

1 Anthropological research indicates that smiles often involve upturned lip corners (Darwin and Prodger [1872] 1998; Schmidt and Cohn 2001).
